# A Control-Theoretic Model of Atherosclerosis

**DOI:** 10.3390/ijms20030785

**Published:** 2019-02-12

**Authors:** Dorota Formanowicz, Jacek B. Krawczyk, Bartłomiej Perek, Piotr Formanowicz

**Affiliations:** 1 Department of Clinical Biochemistry and Laboratory Medicine, Poznan University of Medical Sciences, 61-701 Poznan, Poland; doforman@ump.edu.pl; 2 College of Science and Engineering Flinders University, Adelaide, SA 5042, Australia; jacek.krawczyk@flinders.edu.au; 3 Department of Cardiac Surgery and Transplantology, Poznan University of Medical Sciences, 61-701 Poznan, Poland; bperek@ump.edu.pl; 4 Institute of Computing Science, Poznan University of Technology, 60-965 Poznan, Poland; 5 Institute of Bioorganic Chemistry, Polish Academy of Sciences, 61-704 Poznan, Poland

**Keywords:** atherosclerosis, statins, control-theoretic model, logistic growth

## Abstract

We propose a control-theoretic aggregate model of the progression of atherosclerosis plaque, a chronic inflammatory disease of the arterial wall, to study the basic features of this disease. In the model, we exploit the role of inflammation in the disease progression, and use statins—drugs commonly recommended in atherosclerosis—to control this progression. We use a logistic function to allow for constrained growth of plaque. In the model, both the patient’s age and overall health impact the plaque growth and its sensitivity to statins. The model parameters are estimated using original data, or calibrated using published research as well as our own clinical and laboratory studies. We contend that our model helps to gauge the statins’ impact on a patient’s plaque thickness, hence the disease’s progression and cardiovascular risk, without requiring artery scans.

## 1. Introduction

The aim of this paper is to build and explore a mathematical aggregate model of the progression of atherosclerotic plaque—a dynamic structure undergoing a continual cycle of erosion and repair. Atherosclerosis is treated as a chronic inflammatory disease of the arterial wall, which amounts to deposition of atheromatous plaque on the innermost layer of the walls of large and medium-sized arteries. To capture the basic features of the mentioned phenomena we propose a mathematical model in non-linear non-stationary differential equations, with therapy representing a control variable. We will show how our model can help the clinician to understand the disease development, and to prognosticate the patient’s survival.

There exist mathematical models of the formation of atherosclerotic plaque, see [1,2,3]. They focus mainly on the changes of the local blood flow dynamics in arteries with atherosclerotic lesions. Other researchers, e.g., [4], have created one- and two-dimensional mathematical models to describe selected processes such as recruitment of monocytes into sub-endothelial space. Yet another team of researchers, see [5], created a mathematical model focusing on the low-density lipoprotein cholesterol (LDL-C) and high-density lipoprotein cholesterol (HDL-C) in blood. They showed, by simulations, how the cholesterol profile determines whether plaque will grow or decrease. These scientists also extended their model by including the effect of reverse cholesterol transport and drugs that are used in mice, and conjecture that it may also slow down atherosclerosis progression in humans. Moreover, they included (in [6]) the effects of oxidative disturbances, high blood pressure and smoking in their model. There are also studies that have taken into account the inflammatory aspects of atherosclerosis, similar to those that are included in our work. In particular, Ref. [7] proposed a model of the chemotactic flux of macrophages, in response to cytokines and modified LDL-C; and, Ref. [8] concluded that the interactions between inflammatory cells inside the plaque are responsible for the harmful effect on the plaque composition.

These approaches help us to understand the dynamics of the processes that contribute to atherosclerotic plaque formation at the *micro* level. In contrast to the above-mentioned models, our model is a *macro* model. This is so because we study how a patient’s holistic well-being, controlled by statins, relates to atherosclerosis. Furthermore, a single feature that makes our model significantly different from the models commented on above is that it includes a *control variable* whereby we use this term in the *control-theoretic* sense.

Our model deals with a group of patients, rather than a particular patient’s disease history, for which the model parameters have been estimated or calibrated (where *calibration* refers to assigning values to model parameters based on some micro-evidence or long-run growth facts, see e.g., [9], in the absence of original data). Our outcomes, and calibrated parameters, concur with other models’ graphs and formulae; see in particular [10,11,12,13]. This supports our assertion that our model can help clinicians to gauge the disease’s progression.

Finally, the dynamic model we propose in this paper will be used in our future research. We want to establish, from which stages of atherosclerosis, the disease can be slowed down or brought to a halt, and from which ones it cannot. This will be achieved by framing the atherosclerosis process in *viability theory*—a mathematical theory of constrained dynamic systems, see e.g., [14] or [15].

Here is a brief outline of what the paper contains. We first review the medical background of atherosclerosis. Then we propose a simple mathematical model that captures the basic features of atherosclerosis progression, with particular emphasis on the role of inflammation; we refer to Equation (3) in Section 4.3 (page 8) that explores a proposed link between inflammation and plaque thickness. Next, we consider plaque sensitivity to statin therapy, where statins can control the plaque thickness and stability. A comprehensive mathematical model follows; we refer to Figure 7 in Section 6 (page 13), for an illustration of statins impact on plaque thickness. Concluding remarks summarize our findings. The paper ends with two appendices. In the first, we publish select clinical data obtained in our own study (see the study aggregate results in [16]); in the second, we briefly explain how we have estimated parameters of our main logistic model.

## 2. Medical Background of Atherosclerosis

The pathophysiology of atherogenesis is a very complex and not fully researched process. However, there is a consensus that it progresses through the following main stages:

1. endothelial cell injury;
2. lipoproteins deposition and their oxidation;
3. inflammatory process;
4. fibrous cap formation.

We contend that our aggregate (macro) model captures this process’ consequences, observed in a patient.

Below, we review the main (micro) features of atherogenesis, which we propose are amenable to aggregation in our model.

According to the *response-to-injury theory*, see [17], which has widespread acceptance among medical scholars, the injury to the endothelial cells that line all blood vessels and provide an active, dynamic interface between the blood stream and the arterial wall, causes an inflammatory response which becomes the driving force for the entire process of atherogenesis, see [18].

The factors which may lead to endothelial damage may be any of the agents or processes, individually or in combination, which can cause dysfunction of the cells. A dysfunctional endothelium, directly involved in atherosclerosis, begin to produce cell-surface adhesion molecules, resulting in monocytes and T-lymphocytes to adhere to the endothelium and migrate into the intima. Here, monocytes differentiate into macrophages and begin to accumulate LDL-C, via receptor-mediated phagocytosis and pinocytosis.

According to the *oxidative modification hypothesis*, LDL-C in its native state is not atherogenic, see [19]. The most plausible and biologically relevant modification of LDL is its oxidation, being a hallmark of atherosclerosis, see [20]. As the LDL-C accumulates, their lipids and proteins undergo subsequent oxidation and glycation and form oxidized LDL lipoprotein (ox-LDL), see [21].

On the other hand, there is another hypothesis—*oxidative response to inflammation*—which helps to reconcile the available evidence for oxidative events and vascular disease. According to it, the injurious response to cardiovascular (CV) risk factors is manifested as an inflammatory response in the vascular wall subsequent to lipoproteins retention and vascular injury. This response does not mediate atherosclerosis, but is a by-product of the inflammatory process, see [19].

In the review published in Nature Medicine hypercholesterolemia and inflammatory process were described as “partners in crime”, see [22]. However, the authors of [23] report that none of the lipids ratios have been found to be informative in prediction of acute myocardial infarction (AMI). They then suggest that reduction in serum cholesterol does not prevent the risk of AMI. This is the main reason that between the two above hypotheses, we find the latter more compatible with literature and our experience. In our model, we will use inflammation, rather than LDL oxidation, as an explanatory variable. Here, we also point to [24] whose authors report a data conflict regarding the precise nature of the relationship between LDL and CV, especially in elderly persons. Many studies have reported this relationship as U-shaped [25,26], J-shaped [27], linear [28] and even its lack (see [29,30]) between serum cholesterol level and cardiac events in the elderly [31]. In fact, in some cohorts, mortality due to CV was the highest in the lowest LDL-C quartile [32]).

Typical atheromatous plaque, which is a result of the complex cellular interactions in the intima of the arteries, consists of three major layers: the cellular component that is comprised predominately of vascular smooth muscle cells (VSMC) and macrophages (a fibrous cap); the connective tissue matrix with extracellular lipids; intracellular lipids that accumulate within macrophages (central lipid core rich in lipid-laden macrophages), see [33].

In the established atherosclerotic lesions, activated macrophages accumulate in the shoulder region of atherosclerotic plaque where its cap joins the almost normal vessel wall and starts destabilizing it, directly promoting plaque’s rupture, see [34]. The rupture permits contact between blood and the highly thrombogenic material located in the lesion’s lipid core, e.g., tissue factors, see [35].

The clinical consequences of advanced atherosclerosis in most medium-sized arteries are due to either their progressive lumen encroachment (concentric lesions) or acute plaque’s rupture, usually of a precursor lesion known as vulnerable plaque or thin-cap fibroatheroma, see [36], followed by occluding thrombus formation (eccentric lesions). Although both processes can lead to complete cessation of tissue perfusion, clinical manifestation of this phenomenon is usually different.

Given the importance of the vessel lumen for blood circulation and the plaque role in diminishing it, we will treat carotid intima-media thickness as a model variable, which—if large—identifies high CV risk in individuals (even if the more traditional risk factors might suggest low risk).

## 3. Selection of Variables Included in Our Model

To create an aggregate model of progression of atherosclerosis we have chosen:

- *carotid intima-media thickness*, IMT, as a *state* variable,
- the high-sensitivity level of C-reactive protein, hsCRP, as an explanatory, or *observation* variable (or, output, in the parlance of control theory), and
- a daily dose of statins (normalized to Atorvastatin) as a *control* variable.

Here, we provide a brief justification for that selection.

### 3.1. IMT

According to several large studies, such as the Atherosclerosis Risk in Communities (ARIC), the Cardiovascular Health Study (CHS), and the Rotterdam Study, a correlation between plaque deposits measured as IMT and risk of CV events has been firmly established [37]. Plaque deposition is a *dynamic* process in that the current plaque thickness depends on the previous stage’s thickness. Because of these dynamics, plaque deposits will be a *state variable* in our control-theoretic model. In a clinical setting, *carotid intima-media thickness* (IMT), which depends directly on the deposits, is measured by B-mode ultrasound and treated as a proxy for both plaque advancement and the patient’s survival. (We note  *plaque volume* (PV) has been also measured, see [38]; however, we will use IMT as a widely accepted imaging surrogate marker of generalized atherosclerosis [39]).

### 3.2. hsCRP

As we have explained in Section 2, inflammation plays a central role in all atherosclerosis phases from the initial recruitment of circulating monocytes to the arterial wall to the rupture of unstable atherosclerotic plaque, see [40,41]. Consider the C-reactive protein (CRP), the classical acute phase reactant, which can be measured with high-sensitivity (hs) assays (hsCRP). This blood biomarker has been confirmed to be related to the adverse CV outcomes, e.g., AMI, even in the absence of hyperlipidemia, see [42]. Moreover, it was found that elevated levels of hsCRP may be associated with the presence of macrophages and T-lymphocytes in the plaque, which contributes to its instability and leads to the development of ischemic events [43]. The large JUPITER trial has confirmed these findings in primary prevention in patients with elevated hsCRP but normal LDL-C levels [44]. Moreover, a positive correlation was observed between hsCRP and coronary plaque area, suggesting existence of an important link between hsCRP and coronary inflammation, see [23]. In another study, higher baseline hsCRP level was associated with 12-month all-cause mortality, independent of other prognostic markers, in overweight or obese AMI patients, see [45]. Furthermore, the JUPITER data provide confirmatory evidence regarding the stability of hsCRP levels over time [44]. As a consequence of these arguments, also discussed in Section 2, we have included patient’s hsCRP levels in our model, as an explanatory variable.

### 3.3. Statins

As any inflammation process, the artery inflammation can be controlled or even partially reverted. Statins can exert anti-inflammatory properties, improve endothelial function, increase the bioavailability of nitric oxide, increase antioxidant activity and stabilize plaque by a wide range of mechanisms [46]. Some recent experimental and clinical data suggest that reducing inflammation without affecting lipid levels may reduce the risk of cardiovascular disease [47]. We have decided to use statins as a healing agent, or a *control variable* in our control-theoretic model.

In particular, the JUPITER trial (see [44,48]) confirmed that men and women with elevated hsCRP and low LDL-C are at substantial vascular risk, and demonstrated that statin therapy can cut that risk by half [44]. In [49,50], beyond lipid lowering, statins were found to possess anti-inflammatory properties, evidenced by reduced hsCRP concentration. Overall, the benefits of statins therapy occur primarily in patients with elevated hsCRP [51]. Moreover, the JUPITER trial strongly suggested that elevated hsCRP levels, rather than other factors, are responsible for the high background event rates observed in the trial, despite very low LDL cholesterol levels [44]. (So, normal hsCRP levels might correspond to thinner or more stable plaque). All the above convinces us that statins, as in real life so in our model, can control progression of atherosclerosis.

## 4. Dynamics of Atherosclerosis

In this section, we propose a mathematical model of atherosclerosis’ dynamics. The main phenomena allowed for in the model are the impact of inflammation on plaque growth, a patient’s age and overall health. We complete the model in Section 6 by including statins as a control variable and hence allowing for plaque sensitivity to statins. The selected model variables, as per Section 3, are popular for diagnosis and prognosis in patients with atherosclerosis. This should help understand numerical results obtained from our model.

### 4.1. Logistic Growth

Progression of carotid plaque is an age and lifestyle depended process.

An initially slow plaque buildup in young patients, succeeded by a relatively fast growth of plaque thickness throughout their middle to advanced years, followed by a no-plaque growth in centenarians (see [10]), suggest to us that IMT follows a logistic growth process. Some quantitative evidence of that can be find in [11], where it is shown that the (percentage) plaque growth is fastest in the middle-aged group, which is typical of an S-shaped curve. We will follow these observations and propose a logistic differential equation as a model for IMT. (Here, we note that logistic differential equations have been used in medicine to model, among others, the growth of tumors, see [52]).

Interesting research on IMT variability has been reported in [53]. Its authors investigated an axisymmetric model of intimal thickening using hyperelasticity theory among rodents. While the proposed concept is notable, we have not conducted such detailed analyzes of the variability of selected parameters, and we have focused exclusively on results from human studies.

Mathematically, we propose that the IMT buildup follows a process x(t),t∈[0,T], where x(t)>0 and t≤T is a patient’s age. Process x(t) is a solution to the following logistic differential equation:

(1) $$\frac{dx}{dt}=ax\left(1-\frac{x}{c}\right)\;\;x\left(0\right)=x_{0}\;.$$

Here a>0,c>0 and $x_{0}$ is (very) small and positive. The parameter *a* determines the speed of plaque buildup and can be gender (or race, or lifestyle, or BMI, etc.) specific. The other parameter, *c*—called “carrying capacity” in population dynamics—is determined by the health status of the patient and represents the maximum size of plaque when they pass away. Naturally, $c\in [c_{H},\;{\mathrm{IMT}}_{\max}]$, where $c_{H}>0$ is the *minimum* IMT value, typical of the cardiovascular disease *non-sufferers* (“healthy”) when they are 90 or over; IMT${}_{\max}$ is the plaque’s thickness measured in the disease *sufferers* at the end of their lives. In particular, patients whose medical condition requires dialysis will have a large *c*. Clinical evidence [12,13] suggests that $c_{H}$ can be approximated by 1 mm and IMT${}_{\max}\approx 1.8$ mm.

We contend that as the disease progresses, or recedes, a patient’s value of *c* will vary. Specifically, therapy and/or lifestyle modifications can affect this value.

**Figure 1:**
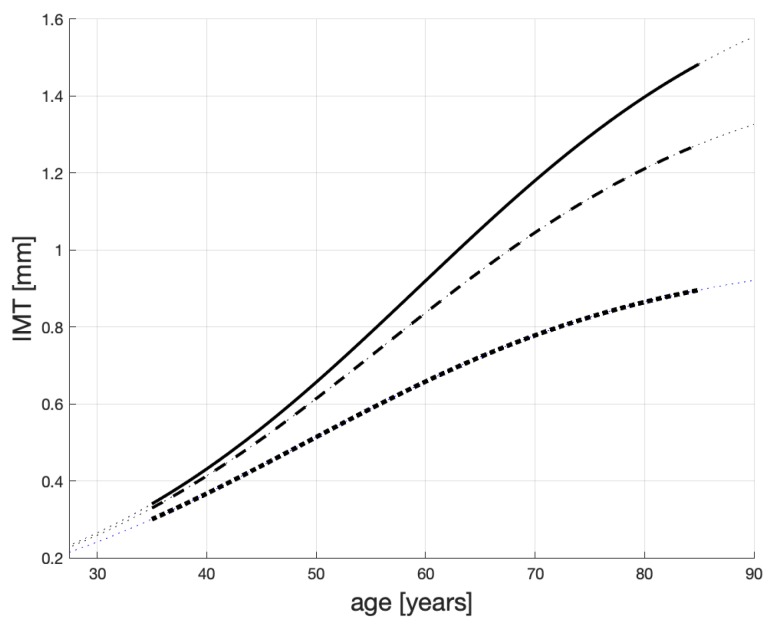
Possible IMT time profiles in persons with different atherosclerosis status. The dashed line represents atherosclerosis non-sufferers; the solid line—non-healthy but not necessarily “heavy” sufferers; the solid line—“heavy” sufferers.

Three possible solutions to Equation (1) are shown in Figure 1. Here, we comment on their general characteristics. We provide some numerical evidence for the shape of these profiles in an Appendix on page 16. An analytical solution to Equation (1) is provided in Equation (A1) on page 17.

Overall, the IMT levels are confined to [0,  1.8]. The dotted line is for atherosclerosis non-sufferers and their $c=c_{H}=1$; a=0.06 and $x_{0}=0.05$. This means that when a healthy patient is 40, their IMT is about 0.37 mm and progresses slowly (approximately 0.015 mm/yr between they are 40 and 60 years old). These patients may live above 100 years, like the healthy Japanese from [10], and die from different causes. For “heavy” sufferers e.g., patients on dialysis, see the solid line, $c={\mathrm{IMT}}_{\max}=1.8,a=0.06$ and $x_{0}=0.05$. Their plaque grows approximately 0.025 mm/yr between they are 40 and 60 years old.

The dashed line represents the IMT progression for patients, who are not healthy but not necessarily are “heavy” sufferers. Their plaque increases approximately 0.021 mm/yr between they are 40 and 60 years old and their IMT may reach 1.5 mm. The other parameters are as for the other lines. We contend that the speeds of the IMT growth reported above, and shown in Figure 1, correspond to those reported in [10,54,55]. In particular, the progression of IMT is documented to accelerate exponentially with age to the eighth decade of life, and slowing in the ninth decade of life (see [54,55]). Another study, [10], proposes a linear correlation between IMT and age, which we think cannot apply to the full age domain. To us, all this points to a logistic model.

In simple terms we propose that the IMT progression depends mainly on $c\in [c_{H},\;{\mathrm{IMT}}_{\max}]$, the parameter that “pulls up” each IMT curve in Figure 1. Should a patient with a large *c* receive treatment or change their lifestyle, the parameter *c* can decrease and the “pull-up” level will diminish. As a result, the growth of IMT will be slower and the patient may live longer.

### 4.2. The Impact of Inflammation

In our view, the level of a patient’s maximum plaque thickness—${\mathrm{IMT}}_{\max}$—i.e., the value toward which the plaque thickness converges, can be explained by this patient’s overall *health level*. For a example, if the *body mass index* (BMI) of a patient starts growing, then this patient’s ${\mathrm{IMT}}_{\max}$ will also grow. If so, the resulting IMT life-span profile—x(t)—will fall somewhere in-between the dotted and solid lines drawn in Figure 1, where—as explained before—the solid line represents the worst case patients’ IMT progression and the dotted line corresponds to that of healthy patients.

A similar result will be brought about by an increasing level of CRP, frequently measured in cardiology clinics as hsCRP. According to [56,57,58], hsCRP is widely recognized as a predictor of atherosclerosis-related cardiovascular morbidity, see Section 2. Moreover, it has been revealed [59] that CRP, a biomarker of low-grade inflammation, may itself contribute to the development of atherosclerotic lesions and to the subsequent acute cardiovascular events. In particular, the view is held that the low-grade local inflammation, manifested by increased levels of hsCRP, accompanies, and aggravates, atherosclerotic plaque buildup. However, many inflammation processes can be effectively managed or completely cured by an appropriate treatment.

We will proxy patient’s health level in year $t\in [t_{1},\;t_{2}]$ by the concentration of hsCRP measured in their blood in that year. (It will be an average value of hsCRP, if several samples were taken. However, the JUPITER data provide confirmatory evidence regarding relative stability of the hsCRP levels over time [44]). Here, the interval $\left[t_{1},\;t_{2}\right]$, $0<t_{1}<t_{2}\leq T$, comprises a patient’s age relevant for our study that covers the period from about 35 until 85 yoa. Given that the plaque thickness depends on patient’s health level, we propose to use the measured amount of hsCRP as an explanatory variable for the patient’s maximum plaque thickness—${\mathrm{IMT}}_{\max}$. In other words, we envisage a mapping, *f*, that maps *C*, the domain of hsCRP, into *Y*, the range of ${\mathrm{IMT}}_{\max}$.

The level of hsCRP, which we denote by *y*, varies in time (e.g., due to therapy) and so will the patient’s ${\mathrm{IMT}}_{\max}$. Therefore, the parameter *c*—the “pulling-up” level in Equation (1) (also see Figure 1)—is, in fact, a time-dependent process $c\left(t\right),\;t\in \left[t_{1},\;t_{2}\right]$. We postulate that the patient’s c(·) is a function of y(·), both assessed at the same time *t*. (Here, we need to mention that the classic version of Equation (1) requires constant parameters. Consequently, if c(·) is a function, we cannot refer to such an equation as *logistic*. Nevertheless, if this function is bounded and slow moving, the resulting solution to an equation like Equation (1) with c(·), will be S-shaped. We will use the term *logistic* to refer to the S-shape.) Certainly, an improvement (lowering) of c(t) will be observed several weeks after therapy has started. However, for simplicity, we assume that the impact of hsCRP on IMT can be observed within the same year and so we drop the time argument on *y* and *c*.

The functional relationship between those two variables should be such that the very low levels of hsCRP are mapped into *c* which is close to $c_{H}$, while the bad health status represented by elevated hsCRP should imply *c* tending to IMT${}_{\max}$.

We suggest the following quantitative relations. The levels of *y*:

- above 1 mg/L indicate an increased risk of CV disease, so the corresponding *c* will have to be greater than $c_{H}=1$;
- between 3–10 mg/L predict a *high* risk of the disease, so the corresponding *c* should approach IMT${}_{\max}$;
- greater than 10 mg/L (and also y>>10 mg/L) indicate acute inflammation unrelated to atherosclerosis and fall out of the scope of our model.

Let C,Y be closed sets such that y∈Y=[0,10] and $c\in C=[c_{H},\;{\mathrm{IMT}}_{\max}]$. The mapping which we propose is f:Y↦C (defined below through Equation (2)). As said above, for *y* ranging between 0 and 1, *f* should be relatively “flat” with the values of *c* close to 1 because it needs to assign values of *c* that characterize healthy patients. For y>1, the maximum IMT levels should increase rather sharply until *c* saturates at the plaque sizes that are equal, or close, to ${\mathrm{IMT}}_{\max}$—see the solid and dashed lines in Figure 1.

The above suggests a logistic profile for the mapping *f*. However, its required sharp increase for y>1 combined with the flat part of *c* for *y* approaching 10 indicates that using inverse tangent (i.e., arctan) may be a better option than using a logistic function. We therefore propose the following mathematical relationship between *y* and *c*:

(2) c=δ+αarctan(y-β).

**Figure 2:**
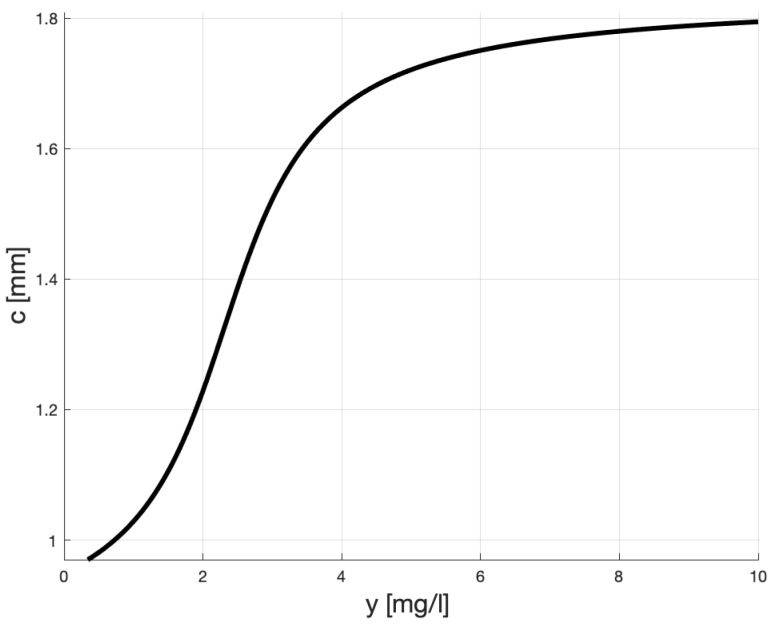
Possible relationship between *y* (hsCRP) and *c* for non-medicated patients.

To calibrate the mapping *f* we need to remember that δ+αarctan(10-β) approximates the largest plaque thickness, here assumed 1.8 and that δ+αarctan(1-β) should be close to 1. The sole value of δ represents some nominal terminal plaque thickness, which can be augmented (respectively, diminished) by non-healthy levels of hsCRP (respectively, healthy). Figure 2 shows the proposed relationship between *y* and *c* which by and large fulfills these conditions. Here, α=0.3242, β=2.313 and δ=1.327.

We can see that in our model, the “pulling-up” plaque level for healthy patients (i.e., those whose y≤1) is less than 1.03 mm, and for heavy atherosclerosis sufferers (i.e., those with 3≤y≤10) is between 1.5 and 1.8. These values are very close to the (right) asymptotes of, respectively, the dotted and solid lines in Figure 1.

Quantitatively, according to our model, non-medicated patients whose hsCRP (i.e., *y*) is 3 mg/L should expect their life-time IMT to grow up to 1.52 mm, which is 84% of 1.8 . For very high levels of *y* (i.e., y≥6) the thickness of plaque is close to IMT${}_{\max}$ = 1.8 mm. This, by and large, fulfills the requirements for *f*, which we have specified above.

### 4.3. Inflammation (hsCRP) Explains Plausible Patient IMT Profiles

Notwithstanding any further benefits from using our model to be discussed in the following sections, we can now say that it may help estimate the *current* IMT level in a patient whose age and hsCRP are known to the physician, *without* requiring an artery scan. For example, consider a 40 year-old male patient whose hsCRP is 4 mg/L. According to Equation (2)

(3) $$\left\{\begin{array}{c} c=1.327+0.3242\arctan(4-2.313)=1.663\\ \mathrm{then}\;\;\mathrm{solve}\;\mathrm{Equation}\;\left(1\right)\;\;\mathrm{with}\;\;c=1.663,a=0.06,x_{0}=0.05\;\left(e.g.,\;\mathrm{use}\;\mathrm{Equation}\;(A1)\right)\\ \mathrm{then}\;\;\mathrm{read}\;\;\mathrm{IMT}(40)=0.423 \end{array}\right.$$

This value is very close to IMT(40) read on the solid line in Figure 1, which is 0.431 (obtained for c=1.8).

The above Formula (2) and procedure (3) provide the clinician with information about a patient’s IMT based on a simple blood test, which includes hsCRP. Otherwise, the (exact) IMT measure is available through an expensive scan.

We notice that the above reasoning can be tested on patients for whom both hsCRP and IMT measurements are available. We hope to conduct such tests in the near future.

Now, we present three hypothetical scenarios of patient health levels. We then show that when these scenarios are fed into, first, Equation (2) and, subsequently, Equation (1), the obtained IMT profiles resemble those displayed in Figure 1. This supports the proposition that our model can explain plaque progression through an analysis of the inflammation process.

**Figure 3:**
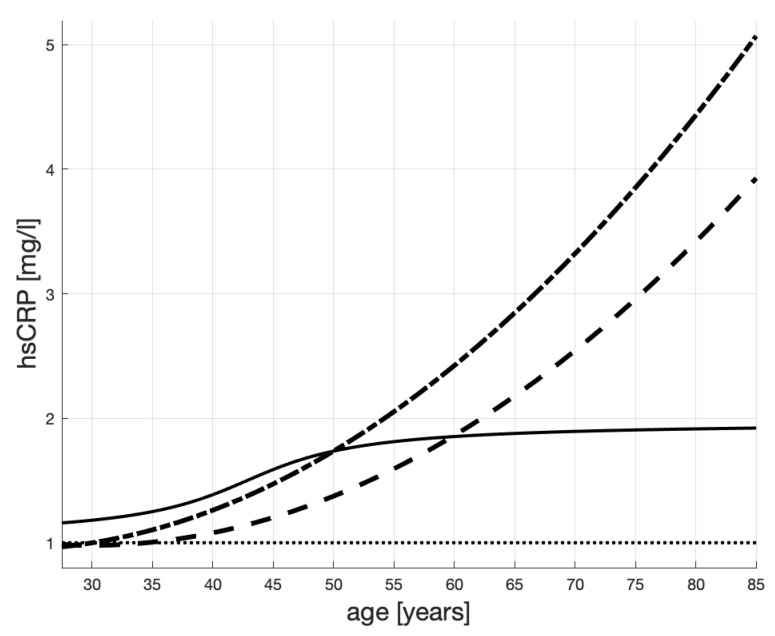
Hypothetical hsCRP scenarios of 3 male patients with different health histories. The dashed line is for a patient who is healthy until his mid-thirties when the levels of hsCRP start growing *slowly*; the dash-dotted line is for a patient who is healthy until his mid-thirties when the levels of hsCRP start growing *rapidly*; the solid line is for a male patient representative of an increased-risk group since young (hsCRP > 1 mg/L) and his health worsens after he turns 30; however, when he is about 45, his hsCRP stabilizes because of lifestyle changes.

The three hypothetical hsCRP (“healthiness”) scenarios are represented in Figure 3:

1. a male patient is healthy until his mid-thirties when the levels of hsCRP start growing slowly—see the dashed line;
2. a male patient is healthy until his mid-thirties when the levels of hsCRP start growing rapidly—see the dash-dotted line;
3. a male patient is in an increased-risk group (hsCRP > 1 mg/L) since young and his healthiness worsens after he turns 30; however, then, because of lifestyle changes, or treatment, his hsCRP stabilizes when he is about 45—see the solid line.

In this figure, we see stabilization of hsCRP in the third patient, which might be a result of treatment. The other patients’ hsCRP profiles are increasing, which might be a result of increasing arterial inflammation.

**Figure 4:**
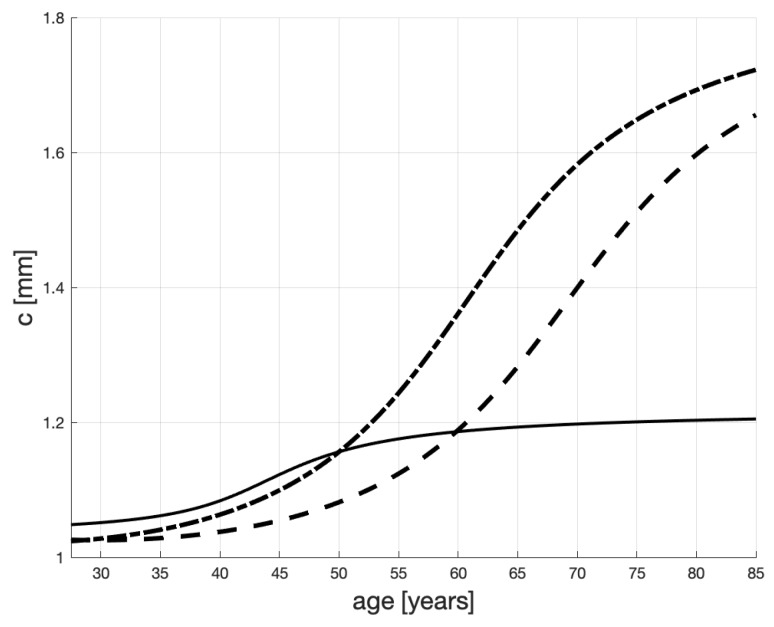
Maximum IMT levels, *c*, as transformed hsCRP scenarios proposed in Figure 3. The dashed line represents a healthy male until his mid-thirties when the levels of hsCRP start growing slowly; the dash-dotted line—a healthy male until his mid-thirties when the levels of hsCRP start growing rapidly; the solid line—a male representing an increased-risk group (hsCRP > 1 mg/L) since young and his healthiness worsens after he turns 30; however, then, due to the proper conduct, his hsCRP stabilizes when he is about 45.

The lines in Figure 4 represent the values of *c*, i.e., the maximum IMT levels (carrying capacity in Equation (1)). These values are obtained by using the mapping (2) to transform the scenarios of hsCRP in Figure 3, into the profiles c(·) in Figure 4. Specifically, we can appreciate how the low level of hsCPR of the third patient—the one who changed his lifestyle (or underwent treatment) at 30—causes stabilization of *c* below 2, which is much below the unhealthy (untreated) patients’ levels.

**Figure 5:**
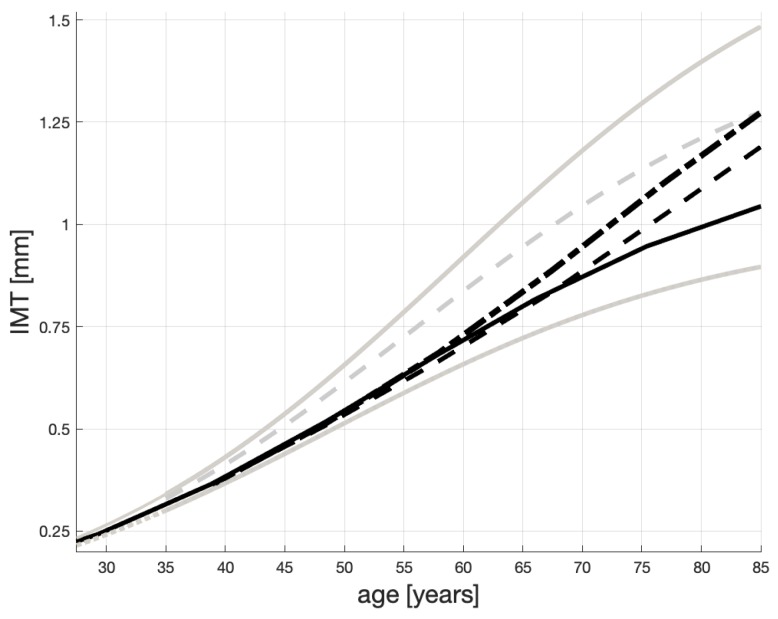
How IMT can evolve given hsCRP age profiles in Figure 3. The gray lines are copied from Figure 1, where the dashed line represents atherosclerosis non-sufferers; the solid line—not healthy but not necessarily “heavy” sufferers; the solid line—“heavy” sufferers. Each evolution is a result of an hsCRP scenario (Figure 3) and, then, of the corresponding progression of the maximum IMT level (Figure 4).

Figure 5 shows the resulting life-span IMT evolutions. (The gray lines are copied from Figure 1.) Each evolution is obtained numerically as a solution to (1), with varying *c* as in Figure 4.

We can now appreciate how complicated the plaque development process can be. e.g., even if the healthiness level of a patient stabilizes, which is pictured by the solid lines in Figure 3 and Figure 4, his (or her) IMT profile is increasing (see the solid line in Figure 5) due to inertia and age progression. While this conclusion may be obvious for clinicians, its visualization and quantification can improve their understanding of the disease dynamics and help communication with patients.

We note that some authors e.g., [10,60] treat the process of thickening of carotid arteries as linear and use linear regression to identify this process. While some of the curves in Figure 5 (and also those in Figure 1) may look straight, they are solutions to non-linear differential equations, which—arguably—capture some of the physical processes responsible for arteries’ thickening better than straight lines.

## 5. Plaque Sensitivity to Statins—3-Hydroxy-3-Methylglutaryl Coenzyme a Reductase Inhibitor

In Section 3 we have spoken of inflammation-reduction properties of statins. Briefly, statins decrease micro-inflammation in the artery’s wall. If so, administration of statins to a patient with an elevated hsCRP (a marker of micro-inflammation—also see Section 3) should reduce its serum concentration and hence stabilize, reduce, or even quell, inflammation. Because of this cause-effect feature, we have selected a daily dose of statins as a control variable in our model.

More in detail, giving statins to patients reduces the macrophages content of atherosclerotic plaque and decreases the activity of the residual macrophage populations. On the other hand, the ox-LDL-induced growth of macrophages and the foam cells formation are also inhibited by statins. Moreover, they have also multiple effects on matrix proteins and collagen. All this is particularly important in the context of the plaque that may rupture easily. Such vulnerable plaque contains high lipids contents, many inflammatory cells, low VSMC and collagen contents, and high levels of VSMC apoptosis, see [61]. All inclusive, the administration of statins should eventually stabilize, or even reduce, the patient’s IMT.

Figure 3, Figure 4 and Figure 5 in Section 4.3 suggest that hsCRP age profiles can have corresponding IMT age profiles. In the current section, we propose a mathematical model of a process through which statins therapy will shape the hsCRP profile. A model like that is the first step to analyze a therapeutic impact of statins on IMT.

According to [48], hsCRP has dropped in their study from 4.2 mg/L to  2.2 mg/L (about 48% of the initial hsCRP) in two years, and to 1.8 mg/L in 4 years (60%), as a result of applying Rosuvastatin 20 mg/day. We will assume that 20 mg/day of Rosuvastatin can be replaced by 80 mg/day of Atorvastatin, see [62,63], and calibrate our model for the latter.

**Figure 6:**
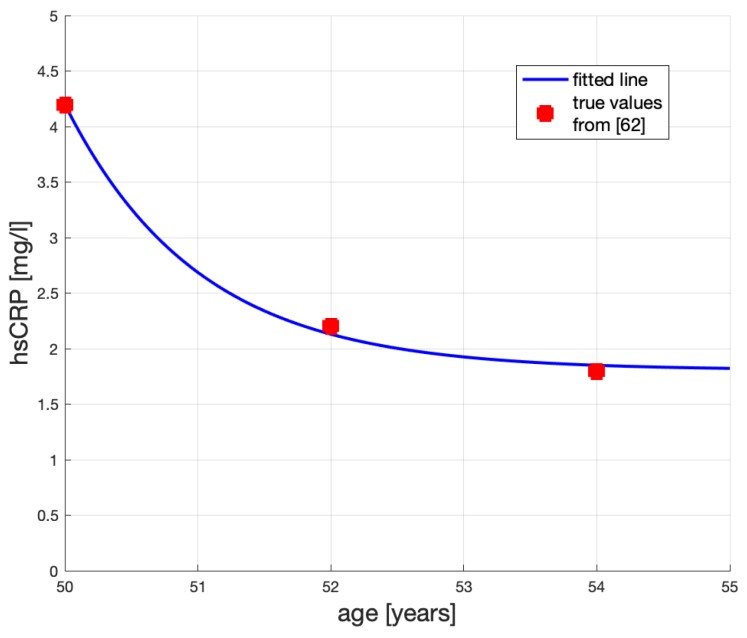
The decreasing effect of statins treatment on hsCRP.

Therefore, as per [48], statins diminish the hsCRP more in the first year of therapy than in the subsequent years. An analytical formula, which allows for diminishing effectiveness, for the healing effect of Atorvastatin *s* (in mg/day) on hsCRP may look as follows:

(4) $$y\left(t\right)=y\left(T\right)\left(1-\sigma \frac{s}{80}\left(1-e^{T-t}\right)\right)$$

where *y* is the measured hsCRP in mg/*ℓ*; *T* is the age at which the patient begins receiving statin, y(T) is his hsCRP score at *T*. Then, t>T and so T-t is the number of years after *T*; σ>0 is a calibrated parameter. Calibrating σ=0.57, to fit model (4) to data from [48], and assuming the male patient’s age is T=50 and his hsCRP is y(50)=4.2 mg/L, yields the hsCRP time profile shown in Figure 6. Here, the large dots represent the measurements reported in [48].

We can see that the exponential function shown in Figure 6 represents quite faithfully what we know about the statin effect: administering statins diminishes hsCRP first quickly and then slowly. Beneficial role of statins in coronary artery disease has been widely discussed in [64]. Based on clinical experience it seems to us that an initial reduction of IMT, in a course of statin treatment, is large because of shrinkage and fibrosis of the plaque. Later, even if the drug dosage does not change, the pace of IMT reduction decreases.

We now want to assess the impact of administering statins on patient IMT.

Decreasing hsCRP by about 30% in four years—illustrated in Figure 6—will affect the “pull-up” level *c* in Equation (1) and will have an impact on the profile of x(t) (IMT) in the years t>T, i.e., after the treatment began at *T*. Clinical evidence [65,66] suggests that the effect of Atorvastatin 80 mg/day on the change of IMT is substantial.

Let us see which results our model based on Equations (1) and (2) predicts for a 50-year-old (“middle-aged”) patient. If the patient is 50, on dialysis (or a candidate for dialysis), male, and his hsCRP = 4.2 mg/L, our model implies their IMT(50)=x(50)=0.6402 mm. i.e., applying Equation (2) yields c=1.6783 mm; then, from Equation (1), we obtain the above value of IMT.

If this was the same patient as in [48], administering Atorvastatin 80 mg/d for two years would lower his hsCRP to y(52)=2.2 mg/L. Then, using (2) yields c=1.2905 mm; solving (1) for this *c* yields x(52)=0.6158 mm. Hence the reduction of IMT is 0.6402-0.6158=0.0244.

Some relevant real-life IMT measurements are reported in [65]. Its authors report a 0.04 mm reduction of IMT among patients aged 45.7±10 with familial hypercholesterolemia, after two years of statin (Simvastatin) treatment. Therefore, our model result is more conservative than the drop reported in [65], but they both are quite close.

However, if we dealt with a 70-year-old patient, male and on dialysis with y(70)=4.2, then our calibrated Formula (4), which does not depend on age, would also predict y(72)=2.2 mg/L, same as for the 52-year-old patient. Because of (2), c=1.6783 as before. For this *c*, Equation (1) yields IMT(70)=1.1276 mm. Next, for y(72)=2.2, we obtain c=1.2905 from Equation (2). For this *c*, solving Equation (1) yields x(72)=0.9703 mm. This suggests the IMT drops by 1.1276-0.9703=0.1573! Unfortunately, no study has confirmed that drop.

Therefore, for older patients, the model (i.e., Equations (1) and (2)) predicts lower ITM than in reality. Furthermore, for patients younger than 45.7±10, the real effect is stronger than the joint application of (2), (1) would have predicted. Therefore, for younger patients, the IMT reduction predicted by our current model will likely underestimate the true reduction, and for older patients—overestimate. This is because patient reaction to Atorvastatin depends on their age. In particular, as atherosclerosis increases with age, the same dosage of statins will have a different effect depending on the patient’s age. We therefore need to generalize Equation (4) to one which allows for patient age. To do so, we will use existing studies, see e.g., [48,67,68,69], which provide real-life observations and findings that affirm and quantify patient age-dependent sensitivity to statins.

In view of the above, we contend that treating a patient with statins (40–80 mg/day) modifies the growth process of IMT in several ways. In particular,

- it stops the plaque growth due to increased fluidness of the plaque building substances;
- it decreases or eliminates inflammation;
- it mobilizes the patient, at least at the beginning of the treatment, to improve their diet and lifestyle, which ameliorate their healthiness.

In brief, it slows down the age-dependent growth of IMT that in our model will be manifested by modulating and decreasing the pull-up level *c*. A mathematical model which accounts for A.–C. is proposed in the following section.

## 6. A Control-Theoretic Model

In this section, we will specify a dynamic relationship between the number of administered statins per day *s* and carotid intima-media thickness x(t) for $T\in [t_{1},t_{2}]$, where *T* is age of the patient at which treatment begins.

Here are the assumptions concerning the control-theoretic model.

- The amount of hsCRP y(T) is measured and known at $T\geq t_{1}$ that is patient’s age at which statins begin to be administered.
- For t<T i.e., before statins are administered, and when a direct measurement of plaque x(t) is *unavailable*, x(t) is a solution to Equation (1) where *c* is determined by (2) and the value of *y* in (2) is the above y(T) i.e., hsCRP measured at time *T*. If x(t) is *available*, *c* could be determined as a solution to (A1), see an appendix on page 17.
- After statins are administered at $T<t_{2}$, the IMT levels $x\left(t\right),t\in \left[T,\;t_{2}\right]$ also follow a solution to (1), but this time *c*—the “pull-up-level” at time *t*—is a time-dependent process c(t), controlled by treatment *s* as follows:
  (5) $$c\left(t\right)=\underset{\mathrm{depends}\;\;\mathrm{on}\;s}{\underset{︸}{\left(x\left(t\right)-\left(\gamma -T\eta \right)\left(1-e^{\frac{s\;\left(T-t\right)}{\Theta }}\right)\;\right)\varepsilon }}+\phi -e^{\varrho \;\left(T-t\right)},\;\;t\in \left[T,t_{2}\right]$$
  where s∈[10,80] [mg/day] is the number of statins given to the patient. Parameters Θ,γ,η,ε,ϕ and ϱ are calibrated. After numerical experiments, we have calibrated these parameters at the following levels: Θ=120,γ=0.6333,η=0.003333,ε=0.65,ϕ=0.75 and ϱ=0.8.
  We note that this time, the solution to Equation (1) will be obtained *numerically* because, with c(t) as in (5), Equation (1) becomes non-stationary and a closed-form solution, like (A1), is unavailable.

Despite its relative complexity, formula (5) can be intuitively explained. First, notice that the exponents of *e* are negative because T<t. Therefore, the longer a patient receives statins, the smaller their incremental (or “marginal”) effect. Moreover, the therapeutic effect of statins diminishes with the patient’s age *T*, at which the statins therapy begins. Evidently, the larger the dose of statins *s*, the stronger their effect. Finally, because of negative exponents the expressions $e^{\mathrm{negative}\;\mathrm{power}\times \mathrm{time}}$ vanish for a large time. This means that there is an upper limit on statins therapy benefits.

The last term of (5) captures a temporary effect, which improved patient’s health can have on *c*, whereas the improvement can be due to mobilization (see C). Constant ε can be interpreted as a weight of improvement in *c* due to the direct statin effect, relative to other causes. Finally, the parameter ϕ is some basic level of plaque thickness.

**Figure 7:**
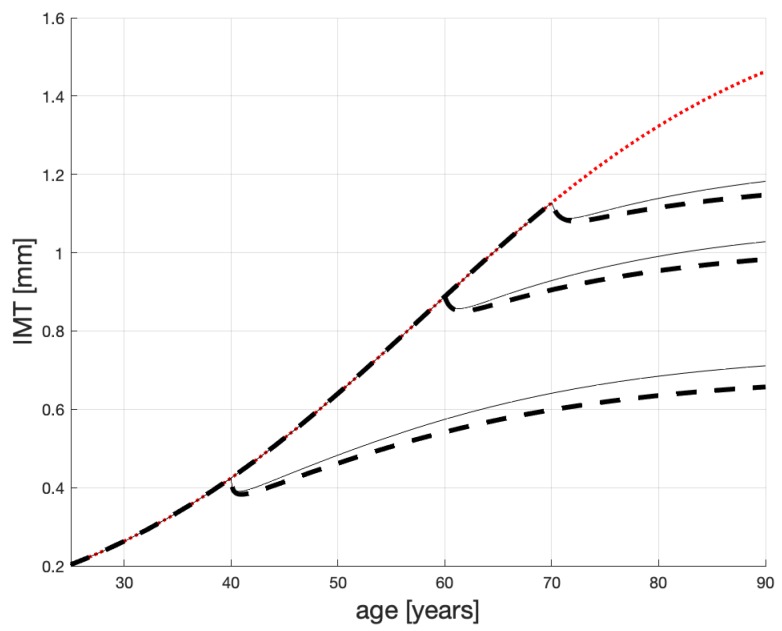
Plausible age profiles for male patients (on dialysis) treated with Atorvastatin: 80 mg/d—dashed line; 40 mg/d—solid line.

The result of using (5) to determine the “pull-up” levels in Equation (1), as a result of administering statin, is presented in Figure 7.

Here, we can see how statins can change the plaque growth. The dotted (red) line represents the plaque progression in an untreated patient. The dashed lines show the impact 80 mg/day of Atorvastatin can have on patients who start treatment when they are, respectively, 40, 60 and 70 years. As in [65] the IMT diminishes by about 0.04 mm in the first 3–4 years. Then, IMT resumes to grow albeit slower than without treatment. The effects of administering 40 mg/day Atorvastatin is shown by the solid (thin black) line.

A general benefit of this figure is in visualizing possible patient’s life-span IMT profiles, sensitivity of these profiles to statins doses and to patient’s age. In particular, this figure, confirms substantial advantages of early administration of statins.

## 7. Concluding Remarks

Atherosclerosis is a very complex disease process with many intermediate stages. A mathematical representation of this disease should help clarify its progression and suggest treatment. We propose that our model fulfills these objectives by allowing the doctor to quantify an expected change in the plaque growth (diminishing, halt or nothing) due to administration of statins.

Graphs obtained from the model show strong resemblance to clinical facts established in the subject literature and to our own clinical and laboratory experience. While, at present, the model parameters have been mainly calibrated, a large clinical study, which we plan to conduct, would produce a large data base. This would allow us to estimate the model parameters, hence improve its accuracy.

Moreover, our model is a necessary starting point for our future research. We will use it to study the atherosclerosis states, from which the disease can be slowed down, or brought to a halt; and from which ones it cannot.

## 8. Methods

Our research consists of mathematical modeling of the dynamic process of atheroscrerosis. By and large we follow a typical approach of control theory to building and applying a dynamic process model. This involves a study of the underlying physical process to ascertain which variables are crucial for this process. The variable which cumulates is IMT and it becomes a state variable. The variable whose amounts can be decided by a doctor, and has an impact on the state variable, is the dose of statins in this case; it becomes a control variable. The one which is observable, and can help assess the impact of statins on IMT is the amount of hsCRP; it becomes an auxiliary variable. Then, mathematical formulae for the relationships between these variables need be proposed and, subsequently, quantified through parameter estimation, or calibrated using literature results, if historical data is unavailable. Finally, the estimated and calibrated formulae are to be used to carry out modeling experiments that might replace medical trials.

## Figures and Tables

**Figure A1:**
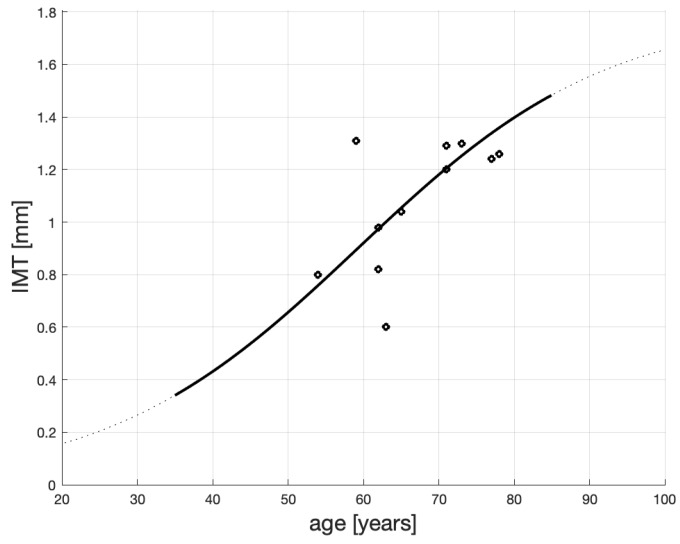
Fitting the IMT time profile for patients on dialysis (severely sick).

**Figure A2:**
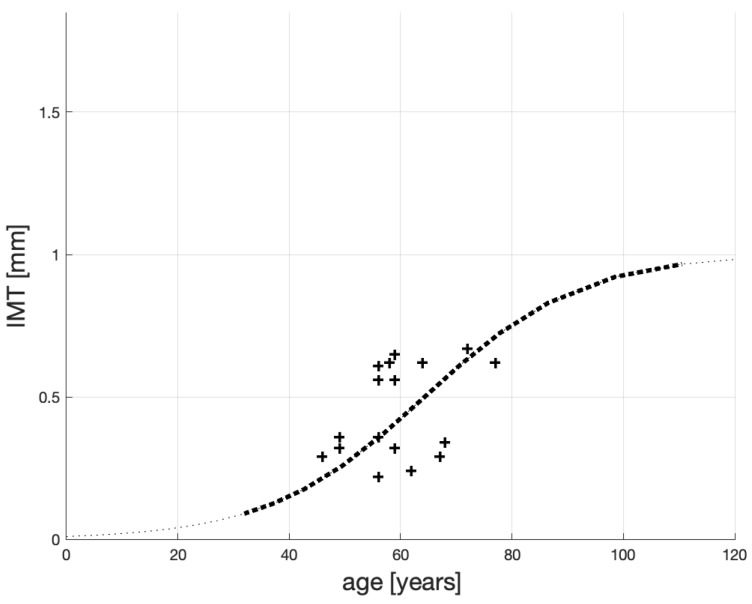
Fitting the IMT time profile for healthy patients.

## Appendix A. Healthy and Hemodialyzed Patients Data

Here, we provide *source* data from a local study, used for estimation of parameters in Equation (1). The study was carried out in accordance with the Declaration of Helsinki of the World Medical Association and approved by the Ethical Committee of Poznan University of Medical Sciences (No 854/14), involved males, aged 40–81 years, treated in various clinics. All qualified participants underwent a careful interview and a clinical examination with an evaluation of patient history based on hospital and outpatient records. The subjects who qualified to the study were monitored and the following disease markers: IMT, hsCRP and eGFR were measured. Data aggregates from this study are published in [16].

- The 20 chronic kidney disease (CKD) patients were assigned to CKD5 group—see Table A1, where the group names are the same as in [16]. This group patients had very severe reduction of kidney function (stage 5th of CKD, estimated glomerular filtration rate (eGFR) <15 mL/min/1.73 $m^{2}$) were hemodialyzed for at least one year, with prescriptions of 4.5–5.5 h/hemodialysis session, 3 times per week. These patients had AMI in the last year and were characterized by particularly complex atherosclerosis. However, persons with diabetes mellitus, an acute inflammatory process (hsCRP > 10 mg/L) and malignant tumors were excluded from this study.
  **Table A1:** Clinical data—hemodialyzed males (CKD5).
  No.	AGE (Years)	IMT (mm)	hsCRP (mg/L)	eGFR (mL/min/1.73 $m^{2}$)	Statin Therapy (Months)
  1	47	0.50	8.25	1.0	0
  2	47	0.55	4.60	1.0	0
  3	47	0.51	4.23	1.0	0
  4	48	1.27	0.82	12.4	0
  5	49	0.54	8.80	0	0
  6	49	0.80	9.37	0	0
  7	54	0.80	5.00	1.0	3
  8	59	1.31	4.44	16.5	9
  9	62	0.82	8.50	0	12
  10	62	0.98	9.30	0	7
  11	63	0.60	8.00	3.0	10
  12	65	1.04	4.32	2.0	8
  13	67	0.43	2.45	11.5	0
  14	71	1.29	5.67	29.0	2
  15	71	1.20	4.68	2.0	4
  16	73	0.62	9.70	4.0	0
  17	73	1.30	5.68	1.0	9
  18	76	0.47	3.21	0	0
  19	77	1.24	6.80	0	4
  20	78	1.26	9.21	8.0	6
  We have used this data to estimate the parameters in Equation (1) for heavy sufferers.
- The HV group, contained healthy males (n=25) with a negative history of acute or chronic inflammation, without any clinical symptoms of atherosclerosis—see Table A2.
  We have used this data to estimate the parameters in Equation (1) for healthy patients.
  **Table A2:** Clinical data—healthy males.
  No.	AGE (Years)	IMT (mm)	hsCRP (mg/L)	eGFR (mL/min/1.73 $m^{2}$)
  1	40	0.41	0.47	104
  2	40	0.55	0.51	95
  3	44	0.56	0.54	79
  4	46	0.49	0.49	105
  5	47	0.63	0.68	88
  6	47	0.64	0.67	107
  7	48	0.53	0.52	100
  8	49	0.39	0.42	91
  9	49	0.57	0.61	92
  10	51	0.52	0.60	63
  11	54	0.52	0.56	91
  12	57	0.63	0.89	107
  13	59	0.54	0.56	73
  14	59	0.76	1.10	74
  15	59	0.63	0.72	113
  16	59	0.59	0	63
  17	60	0.60	0.65	67
  18	60	0.73	1.10	74
  19	61	0.87	1.20	95
  20	66	0.50	0.52	68
  21	67	0.71	1.10	66
  22	68	0.65	0.67	93
  23	68	0.70	0.90	72
  24	70	0.70	0.78	65
  25	71	0.70	1.00	64

## Appendix B. Comments on Parameter Estimation in Equation (1)

A solution to Equation (1) is a logistic function and so we look for parameters that best fit this kind of function.

### Appendix B.1. Parameters in Equation *(1)*

- We have used the men’s data from Table A1 to estimate the parameters in Equation (1) for the severely sick patients. The IMT numbers from Table A1 are represented by the black ‘o’ in Figure A1.
  To create a solution to Equation (1) we have used the MATLAB routine: Curve Fitting Tool. We have set suitable bounds in this routine, on the estimated parameters $a,c,x_{0}$, following the findings on IMT and its growth in [12,70,71]. Using Curve Fitting Too yields $a=0.06,c=1.8,x_{0}=0.05$. The resulting statistics are SE = 0.389, $R^{2}=0.36$, Adjusted $R^{2}=0.2863$. The line in Figure A1 represents the best-fit *logistic* function. This line is the same as the solid line in Figure 1.
- To estimate $a,c,x_{0}$ in Equation (1) for the healthy patients, we have used the mens’ data from Table A2. The IMT measurements are represented by the black ‘+’ in Figure A2.
  To create a solution to Equation (1), we have also used Curve Fitting Tool and set suitable parameter bounds. The routine yielded $a=0.06,c=1,x_{0}=0.05$. The resulting statistics are SE = 0.292, $R^{2}=0.0004$. Note that small or even negative values of $R^{2}$ may occur when fitting non-linear functions to data. Our model is non-linear, and this can explain the obtained value of $R^{2}$. Adjusted $R^{2}=0.0404$. The line in Figure A2 represents the best-fit *logistic* function. This line is the same as the dotted line in Figure 1.

We stress that our aim is not to find an *overall* best fit, but the best fit in a certain class of functions (here *logistic*), which have a clinical interpretation. Nevertheless, the small SSE values and a significantly greater Adjusted $R^{2}$ than $R^{2}$ for the healthy patients add credibility to our model.

### Appendix B.2. An Analytical Solution to Equation *(1)*

The logistic Equation (1) yields the following solution:

(A1) $$x\left(t\right)=\frac{cx_{0}e^{at}}{c+x_{0}\left(e^{at}-1\right)}$$
